# Identification of consensus hairpin loop structure among the negative sense subgenomic RNAs of SARS-CoV-2

**DOI:** 10.1186/s42269-023-01002-3

**Published:** 2023-02-23

**Authors:** Naveen Prakash Bokolia, Ravisekhar Gadepalli

**Affiliations:** Viral Research and Diagnostic Laboratory, Microbiology Department, All India Institute of Medical Sciences, Jodhpur, 342001 India

**Keywords:** SARS-CoV-2, Subgenomic RNA, Secondary structure, Transcriptional pausing, Hairpin loop structure

## Abstract

**Background:**

SARS-CoV-2 is the causative agent of worldwide pandemic disease coronavirus disease 19. SARS-CoV-2 bears positive sense RNA genome that has organized and complex pattern of replication/transcription process including the generation of subgenomic RNAs. Transcription regulatory sequences have important role in the pausing of replication/transcription and generation of subgenomic RNAs.

**Results:**

In the present bioinformatics analysis, a consensus secondary structure was identified among negative sense subgenomic RNAs of SARS-CoV-2. This consensus region is present at the adjacent of initiation codon.

**Conclusions:**

This study proposed that consensus structured domain could involve in mediating the long pausing of replication/transcription complex and responsible for subgenomic RNA production.

**Supplementary Information:**

The online version contains supplementary material available at 10.1186/s42269-023-01002-3.

## Background

The severe acute respiratory syndrome coronavirus type 2 (SARS-CoV-2) that causes the global pandemic illness COVID-19 has a 29.9 kb positive sense single strand RNA genome (Kim et al. 2020; Zhu et al. 2020a). Inside the host environment, the RNA genome goes through a complicated replication/transcription process. In order to translate the viral proteins and package the RNA genome into virion particles, the SARS-CoV-2 enters the host cell and undergoes replication of the positive sense RNA genome. In the process, the negative sense RNA genome is created. The 70–75% genome consists of *ORF1a* and *ORF1b* that encodes for non-structural proteins and remaining ORFs encodes for structural and accessory proteins (Fung and Liu 2021). The structural proteins are spike (S), membrane (M), envelope (E), and nucleocapsid (N) proteins, whereas non-structural proteins are nsp1 to nsp16 (Kumar et al. 2019; Naqvi et al. 2020). SARS-CoV-2 RNA genome bears conserved transcriptional regulatory sequences (TRS) of 6–7 nucleotides (Yang et al. 2020). The TRS is present at the immediate upstream of initiation codon, where replication/transcription complex is paused (Alexandersen et al. 2020; Kim et al. 2020). RNA-dependent RNA polymerase (RdRp) is the nsp-12 that has the important property to backtracking that causes long pausing at specific site (Dulin et al. 2015; Malone et al. 2021). Therefore, in addition to complete RNA genome, a nested set of negative sense subgenomic RNAs is also generated including S subgenomic, 3a subgenomic, E subgenomic, M subgenomic, 6 subgenomic, 7a subgenomic, 7b subgenomic, 8 subgenomic, N subgenomic and 10 subgenomic. These canonical subgenomic RNAs are thought to be generated through the complex mechanism that involves pausing of negative sense RNA synthesis by RNA-dependent RNA polymerase (RdRp) (Alexandersen et al. 2020; Kim et al. 2020; Mohammadi-Dehcheshmeh et al. 2021; Yang et al. 2020).

Although the conserved role of TRS has been identified/proposed in the pausing of transcription, the role of RNA secondary structure has not been identified in this perspective. Therefore, in this study investigation of conserved secondary structure pattern was performed nearby the initiation codon/TRS that might be important in pausing of transcription during negative sense RNA synthesis of the SARS-CoV-2 genome.

The analysis of negative sense subgenomic RNAs was objectively performed in this study because of following important reason. The negative sense RNA is firstly generated through the replication/transcription of positive sense RNA genome. The negative sense RNA will serve as template for positive sense RNA genome and subgenomic messenger RNAs (or subgenomic RNAs) (Sawicki et al. 2007; Yang et al. 2020). According to previous research model, it was suggested that strand exchange or 5′ leader (5′ UTR) sequence to body (RNA) fusion occurs at TRS during negative sense RNA synthesis (Kim et al. 2020). Therefore, the consensus secondary structural motifs nearby the TRS might be important in several perspectives. In the current study, the bioinformatics analysis of negative sense subgenomic RNAs was performed that revealed a consensus hairpin loop secondary structure. Simultaneously, we also validated the findings in parallel comparison with SARS-CoV and MERS-CoV. SARS-CoV-2 genome has 79.5% sequence similarity with SARS-CoV and only 50% homology with MERS-CoV (Lu et al. 2020; Zhu et al. 2020b). Within this context, the present study aims to reveal the specific hairpin domain structure at the adjacent of initiation codon/TRS that is consistently present among subgenomic RNAs of both SARS-CoV-2 and SARS-CoV. This is the novel finding where the consensus secondary structure has been identified within the perspective of replication/transcription of genomic RNA of SARS-CoV-2. This study proposed the novel molecular mechanism of consensus hairpin loop secondary structure that could mediate the transcriptional pausing during the replication of genomic RNA and subsequent generation of subgenomic RNAs.

## Methods

### Retrieval of genomic and subgenomic RNAs sequences of SARS-CoV-2 and SARS-CoV

The complete genome sequence of SARS-CoV-2 isolate Wuhan-Hu-1 (NCBI Reference Sequence: NC_045512.2) was used in this study, and specific regions (upstream and downstream) corresponding to particular subgenomic RNAs were retrieved from NCBI Virus resource portal. The RNA sequences (positive sense) were formatted and converted in to negative sense subgenomic RNAs by using Visual Gene Developer (Jung and McDonald 2011). The negative sense RNAs were used in alignment and secondary structure prediction analysis. To analyze the subgenomic RNAs of SARS-CoV, complete genome sequence of Bat coronavirus (BtCoV/279/2005) was retrieved from NCBI Virus resource portal and subsequently used in analysis.

### RNA secondary structural similarity by using LocARNA webserver

LocARNA-Alignment and Folding webserver align the input RNA sequences and simultaneously fold them (Raden et al. 2018; Will et al. 2012). This webserver was used in the present study because it folds the RNA by using very realistic energy models as used by RNAfold of the Vienna RNA package. The sequences of negative sense subgenomic RNAs were aligned by using LocARNA webserver. The alignment type was global and in standard mode. The alignment was used to identify the consensus secondary structures elements between negative sense subgenomic RNA sequences of SARS-CoV-2.

### Secondary structure prediction by using Vienna RNA webserver

Secondary structure prediction of subgenomic RNA sequences was performed by using Vienna RNA webserver (Hofacker 2003). The subset of aligned sequence region (~ 285 nt) respective to each subgenomic RNA was submitted for structure prediction by using Vienna RNA webserver. The prediction results are included in supplementary data file.

## Results

### The negative sense subgenomic RNAs bear conserved hairpin domain at immediate downstream of initiation codon

Previous research investigation determined the abundance levels of subgenomic RNAs from the COVID-19 patient samples (Alexandersen et al. 2020). Authors mapped the NGS reads data to subgenomic RNAs in order to identify the abundance of particular subgenomic RNA from the patient samples (Alexandersen et al. 2020). NGS reads mapped results revealed that Orf7a and N subgenomic were in high abundance, whereas Orf8, Orf6, and E subgenomic were relatively low (in an increasing order). These subgenomic RNAs are present several fold higher in comparison with whole genome fragments. Therefore, the present study seek to identify the possible conserved regions nearby the TRS or initiation codon which could mediate significant roles in the generation or expression of subgenomic RNAs.

**Fig. 1:**
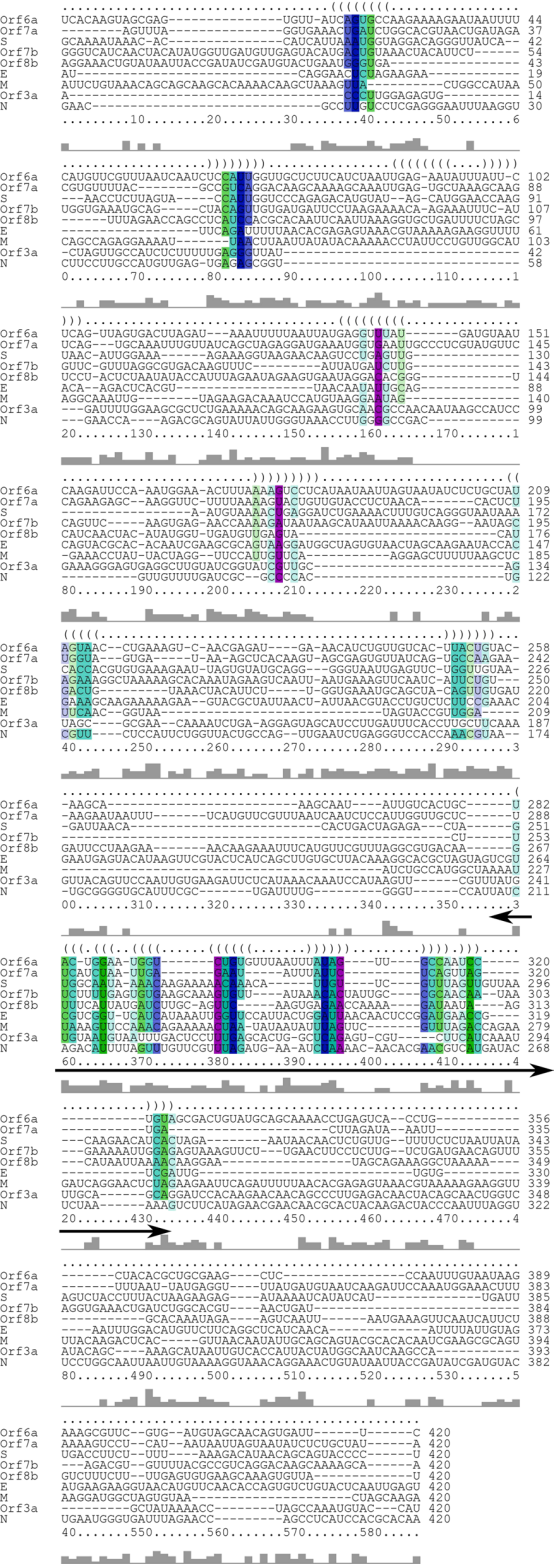
The figure presents the output of alignment result of negative sense subgenomic RNAs of SARS-CoV-2 that was done through the LocARNA webserver. The alignment result revealed the consensus sequence and hairpin structure (with double headed arrow) among negative sense subgenomic RNAs. This consensus structure is present at the immediate downstream of initiation codon or TRS (mentioned with double-headed arrow)

Therefore, in order to identify the consensus secondary structure, negative sense subgenomic RNAs were aligned by using LocARNA webserver. The significant length of upstream and downstream region (with respect to initiation codon) was considered for the analysis, with the total length of ~ 420 nt. Sequence alignment revealed a consensus hairpin domain (~ 35 to 60 nt; depending on the particular subgenomic RNA) among different negative sense subgenomic RNAs (Fig. 1, Table 1). This domain is present at the immediate downstream of initiation codon. The important features could be noted as the distance between initiation codon and consensus hairpin domain (Table 1). The structured hairpin domain of negative sense subgenomic RNAs might have intrinsic functioning and mediate the pausing/backtracking of RdRp at the immediate downstream of initiation codon. The conserved hairpin domain could also have role in template switching during transcription, although the role of conserved TRS has only been identified in recombination events (Yang et al. 2021). The conserved TRS is AACGAAC, which is highlighted in green (as GUUCGUU; negative sense) in the analyzed sequences of negative sense subgenomic RNAs (Additional file 1).

**Table 1:** The description of analyzed negative sense subgenomic RNAs

Sr. No	Type of subgenomic RNA	Location of hairpin structure (from the downstream of initiation codon)	Length of hairpin loop structure with probability	Hairpin structure is distinct or not (merged with different loop carrying initiation codon)	Sequence alignment matches with predicted structure (probability)	Probability of formation of distinct secondary structure
1	S subgenomic	Includes ~ 28 nt of coding region	~ 55 nt	No (merged with initiation codon)	Yes	Less
2	*3a subgenomic	~ 10 nt	~ 58 nt	Yes	Yes	Moderate
3	***E subgenomic	~ 47 nt	~ 52 nt	Yes	Yes	High
4	*M subgenomic	~ 8 nt	Splits in three small hairpin loops	No (first loop merged with initiation codon)	No (three distinct hairpin structures)	Moderate
5	**Orf6 subgenomic	~ 41 nt	~ 47 nt	Yes	Yes	High
6	****Orf7a subgenomic	~ 30 nt	~ 35 nt	Yes	Yes	High
7	Orf7b subgenomic	Includes ~ 6 nt of coding sequence	Splits in two hairpin loop structure	No (first loop merged with initiation codon)	No (two distinct hairpin structures)	Moderate
8	**Orf8 subgenomic	~ 20 nt	~ 57 nt	Yes	Yes	Moderate
9	****N subgenomic	~ 1^st^ nucleotide of initiation codon	~ 60 nt	Yes	Yes	Moderate

The description is drawn from the combined output of sequence alignment followed by secondary structure prediction results. The number of asterisks (*) denotes to the abundance level for each subgenomic RNA detected by NGS reads mapped to the sequences, and no asterisk is given to particular subgenomic that was detected at very low or near zero level according to previous finding (Alexandersen et al. 2020). Single asterisk denotes low, two and three asterisk denotes moderate and four asterisk denotes high abundance of particular subgenomic RNA (in relative context)

### The Orf7a subgenomic, N subgenomic, E subgenomic, Orf6 subgenomic and Orf8 subgenomic bear distinct secondary structure domain near the TRS

**Fig. 2:**
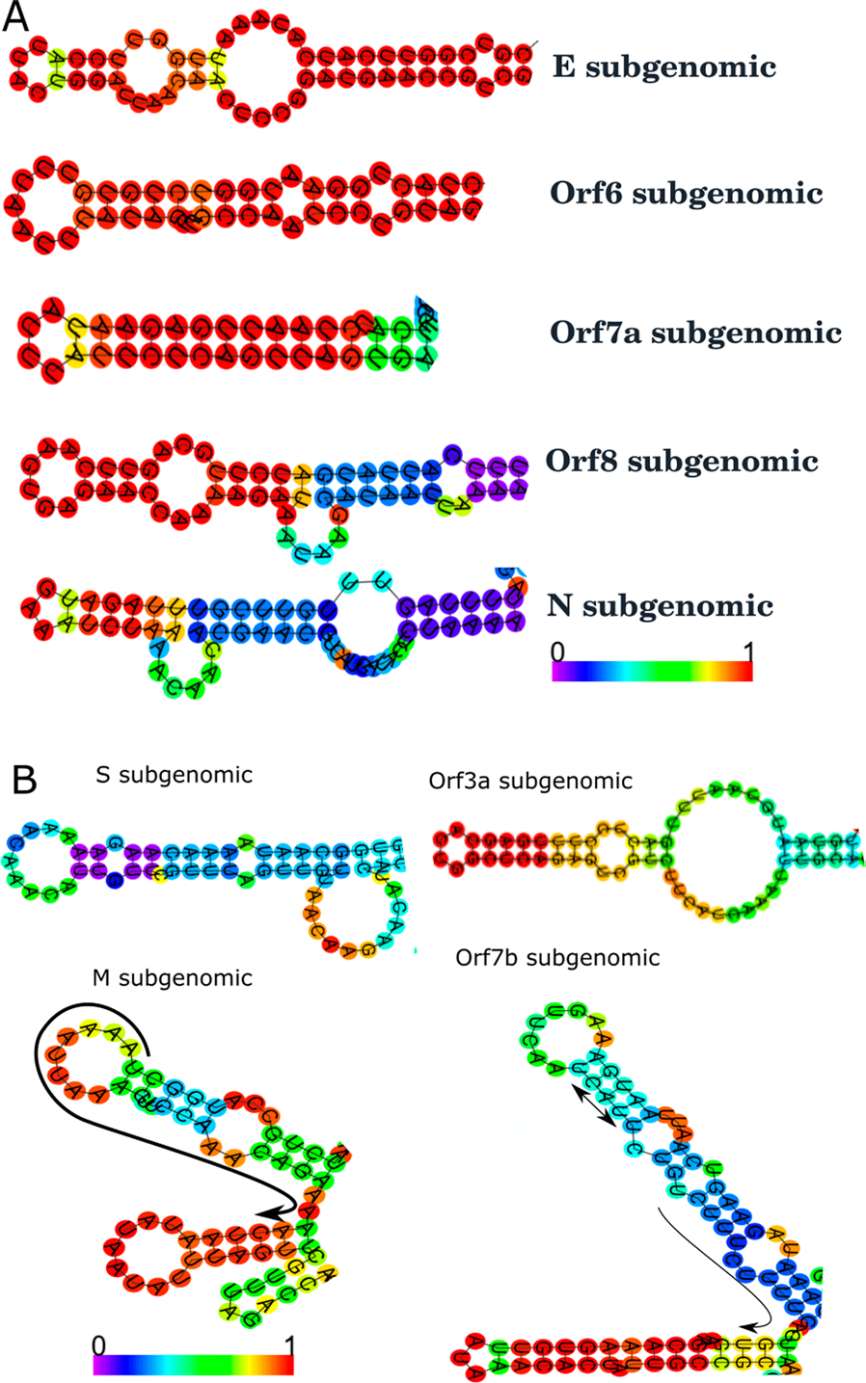
Secondary structure of consensus sequence of respective negative sense subgenomic RNAs. The secondary structure of each sequence was predicted by using Vienna RNA webserver. The respective portion of hairpin loop is shown with probability in color coding from 0 to 1. Secondary structure of consensus alignment sequence of each subgenomic RNA is shown in panel A and B. Panel A involves consensus secondary structure from subgenomic RNAs those have been detected or mapped at considerable level through NGS reads mapping (Alexandersen et al. 2020). Panel B involves consensus structure of subgenomic RNAs those have been detected at very low or zero level through NGS reads mapping. The complete secondary structure of each subgenomic RNA is provided in supplementary data file

**Fig. 3:**
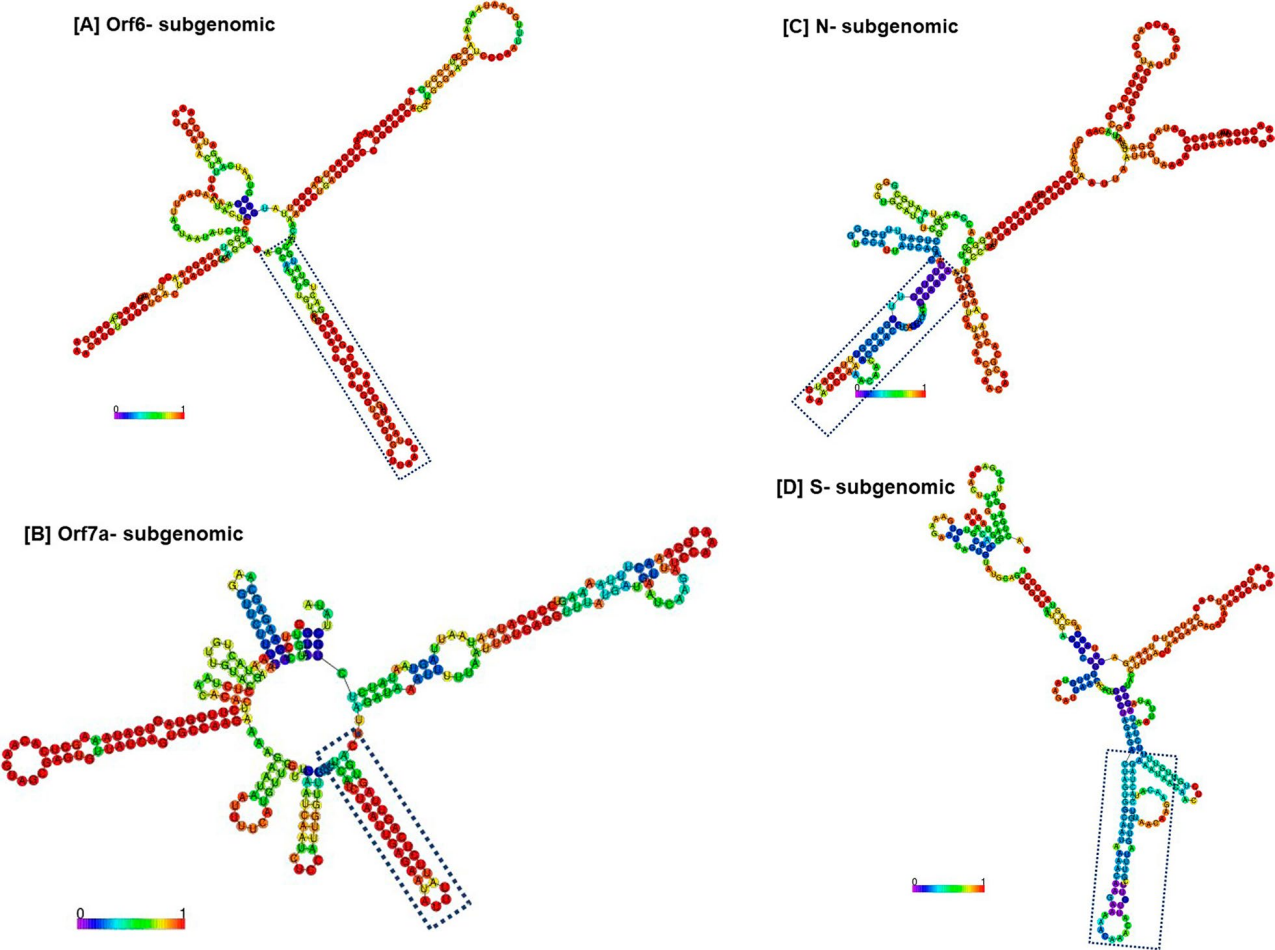
The panel involves the secondary structures of representative subgenomic RNAs. The consensus secondary structure of respective subgenomic RNA (Orf6 subgenomic, Orf7a subgenomic, N subgenomic, and S subgenomic) is shown in a distinct box (dotted blue line)

The sequence alignment of negative sense subgenomic RNAs revealed a single hairpin domain. The probability of hairpin domain formation of subgenomic RNAs was further determined at the level of secondary structure. The secondary structure prediction was performed by using Vienna RNA webserver, and determined whether particular subgenomic RNA adopts a specific hairpin structure or not. Data obtained from sequence alignment and secondary structure prediction indicate that Orf7a subgenomic, N subgenomic, E subgenomic, Orf6 subgenomic and Orf8 subgenomic bear distinct and long hairpin domain with the higher probability (Table 1, Fig. 2A, Fig. 3). These structural features could possibly be correlated with the previous study by Alexandersen et al. 2020, which described the high abundance of Orf7a subgenomic and N subgenomic RNAs, whereas Orf8 subgenomic, Orf6 subgenomic, and E subgenomic were relatively low in abundance (Alexandersen et al. 2020). In addition, the remaining subgenomic RNAs (S subgenomic, Orf7b, Orf3a and M subgenomic) were reported to be very low or near zero level abundance (Alexandersen et al. 2020). The underlying reason of low level of NGS reads could be correlated with their respective hairpin structure (Fig. 2B) and other properties mentioned in Table 1. These features include: hairpin loop splits in to subdomains, hairpin loop merged with initiation codon, length and probability of hairpin domain formation. Overall, the formation of distinct hairpin structure might be the underlying feature in the generation of negative sense subgenomic RNAs.

Importantly, the highest similarity (and intermediate alignment) was observed between Orf7a and N subgenomic RNAs, as both subgenomic RNAs were placed together in guide tree. It provides significant relevance and correlation from the previously reported high abundant reads of N and Orf7a subgenomic (Alexandersen et al. 2020). In this perspective, we propose that conserved secondary structural elements might be a determining factor in transcriptional pausing and backtracking of RdRp that leads to subgenomic RNA production.

### Analysis of consensus secondary structure prediction in SARS-CoV and MERS-CoV subgenomic RNAs

It is important to validate or examine the possibility of consensus secondary structure in closely related enveloped viruses (beta-coronaviruses). Within this context, we further analyzed the subgenomic RNA sequences of SARS-CoV and MERS-CoV. We identified a distinct hairpin structure domain from the sequence alignment of subgenomic RNA sequences of SARS-CoV and MERS-CoV (Additional file 1: Figure S1 and 2). The SARS-CoV and MERS-CoV subgenomic RNAs bear a distinct hairpin domain at the immediate downstream of TRS, as observed in the case of subgenomic RNAs of SARS-CoV-2 (Additional file 1). However, after analysis it was observed that an additional domain could be considered in the case of some of the subgenomic RNAs of SARS-CoV as it falls at the immediate upstream of TRS, whereas for some of the specific subgenomic RNAs the second downstream domain could additionally be considered (as the consensus region falls at the immediate downstream of TRS).

## Discussion

Coronaviruses have conserved sequence of 7–8 nt that has important role in template switching during the replication/transcription of SARS-CoV-2 RNA genome (Kim et al. 2020). In this study, we identified the consensus secondary structure among the negative sense subgenomic RNAs of SARS-CoV-2 that also involve the conserved TRS motif. The consensus secondary structure is present at the adjacent of TRS, therefore, indicates the additional role in transcriptional pausing that could be mediated by specific secondary structure at 5' end. It has been thought or hypothesized that conserved TRS (TRS-B) increases the probability of template switching of RdRp that is mediated by hybridization with identical core sequence in the TRS-L (Sola et al. 2015; Zúñiga et al. 2004). However, additional secondary structure elements could have role in recombination events, and experimental investigations could be made, where the effect of deletion in consensus secondary structure (Fig. 3) could be made, and subsequent impact on the production of subgenomic RNAs could be studied.

Moreover, such secondary structures could also have additional role in halting the exoribonucleases (Xrn2) and maintaining the stability of subgenomic RNAs. Exoribonuclease-resistant RNAs (highly structured) have already identified in flaviviruses and dianthoviruses that prevents the noncoding region from degradation (Pijlman et al. 2008; Steckelberg et al. 2018a, b).

## Conclusions

The present finding revealed the conserved hairpin structure that is present in negative sense subgenomic RNAs of SARS-CoV-2. The significance of conserved hairpin structure could be of twofold reasons. Firstly, the particular hairpin loop secondary structure could have intrinsic functioning in the pausing of replication/transcription of negative sense subgenomic RNAs mediated by RdRp. In addition, the conserved secondary structure could facilitate template switching (by unknown mechanism) at TRS during transcription that involves the joining of 5ʹUTR to RNA body sequence. Further experimental studies could be performed to identify the much precise role of the conserved secondary structure nearby the initiation codon/TRS in the generation of negative sense subgenomic RNAs and maintenance of viral infection.

## Supplementary Information

**Additional file 1: **Title of Data: Negative sense Subgenomic RNAs sequences of SARS-CoV-2 used in this study for alignment. Description of Data: This section includes negative sense subgenomic sequences of SARS-CoV-2 those were used alignment. Title of Data: Negative sense subgenomic RNAs of SARS-CoV. Description of Data: This section includes negative sense subgenomic sequences of SASR-CoV those were used alignment.

## Data Availability

All data generated or analyzed during this study are included in this published article and its supplementary files.

## Abbreviations

SARS-CoV-2: Severe acute respiratory syndrome coronavirus-2
COVID-19: Coronavirus disease-19
RNA: Ribonucleic acid
RdRp: RNA-dependent RNA polymerase
UTR: Un-translated region
Nsp: Non-structural protein
TRS: Transcriptional regulatory sequence
ORF: Open reading frame
MERS-CoV: Middle East respiratory syndrome-related coronavirus
Xrn-2: Exoribonuclease-2
