# Supplementary material for: Identification of consensus hairpin loop structure among the negative sense subgenomic RNAs of SARS-CoV-2
Source: Bull Natl Res Cent. 2023 Feb 23;47(1):28. doi: 10.1186/s42269-023-01002-3 (PMC9947893; doi:10.1186/s42269-023-01002-3)
Supplement: Supplementary file 1 — Additional file 1: Title of Data: Negative sense Subgenomic RNAs sequences of SARS-CoV-2 used in this study for alignment. Description of Data: This section includes negative sense subgenomic sequences of SARS-CoV-2 those were used alignment. Title of Data: Negative sense subgenomic RNAs of SARS-CoV. Description of Data: This section includes negative sense subgenomic sequences of SASR-CoV those were used alignment. [file 42269_2023_1002_MOESM1_ESM.pdf]

Negative sense Subgenomic RNAs sequences of SARS-CoV-2 used in this study for alignment.
Highlighted region: Initiation codon: yellow; TRS: Green; Consensus hairpin structure: Pink

> S gene sub-genomic RNA
GCAAAAUAAACACCAUCAUUAAAUGGUAGGACAGGGUUAUCAAACCUCUUAGUACCAUUGGUCCCAGAG
ACAUGUAUAGCAUGGAACCAAGUAACAUUGGAAAAGAAAGGUAAGAACAAGUCCUGAGUUGAAUGUAA
AACUGAGGAUCUGAAAACUUUGUCAGGGUAAUAAACACCACGUGUGAAAGAAUUAGUGUAUGCAGGGG
GUAAUUGAGUUCUGGUUGUAAGAUUAACACACUGACUAGAGACUAGUGGCAAUAAAACAAGAAAAACA
AACAUUGUUCGUUUAGUUGUUAACAAGAACAUCACUAGAAAUAACAACUCUGUUGUUUUCUCUAAUUA
UAAGUCUACCUUUACUAAGAAGAGAUAAAAUCAUAUCAUUGAUUUGACCUUCUUUUAAAGACAUAACA
GCAGUACCCCU

> Orf3a Subgenomic
ACCCUUGGAGAGUGCUAGUUGCCAUCUCUUUUUGAGGGUUAUGAUUUUGGAAGCGCUCUGAAAAACA
GCAAGAAGUGCAACGCCAACAAUAAGCCAUCCGAAAGGGAGUGAGGCUUGUAUCGGUAUCGUUGCAGU
AGCGCGAACAAAAUCUGAAGGAGUAGCAUCCUUGAUUUCACCUUGCUUCAAAGUUACAGUUCCAAUUG
UGAAGAUUCUCAUAAACAAAUCCAUAAGUUCGUUUAUGUGUAAUGUAAUUUGACUCCUUUGAGCACUG
GCUCAGAGUCGUCUUCAUCAAAUUUGCAGCAGGAUCCACAAGAACAACAGCCCUUGAGACAACUACAGC
AACUGGUCAUACAGCAAAGCAUAAUUGUCACCAUUACUAUGGCAAUCAAGCCAGCUAUAAAACCUAGCC
AAAUGUACCAU

> E gene Subgenomic RNA
AUCAGGAACUCUAGAAGAAUUCAGAUUUUUAACACGAGAGUAAACGUAAAAAGAAGGUUUUACAAGAC
UCACGUUAACAAUAUUGCAGCAGUACGCACACAAUCGAAGCGCAGUAAGGAUGGCUAGUGUAACUAGCA
AGAAUACCACGAAAGCAAGAAAAAGAAGUACGCUAUUAACUAUUAACGUACCUGUCUCUUCCGAAACGA
AUGAGUACAUAAGUUCGUACUCAUCAGCUUGUGCUUACAAAGGCACGCUAGUAGUCGUCGUCGGUUCA
UCAUAAAUUGGUUCCAUUACUGGAUUAACAACUCCGGAUGAACCGUCGAUUGUGUGAAUUUGGACAUG
UUCUUCAGGCUCAUCAACAAUUUUAUUGUAGAUGAAGAAGGUAACAUGUUCAACACCAGUGUCUGUAC
UCAAUUGAGU

> M gene SUbgenomic RNA
AUUCUGUAAACAGCAGCAAGCACAAAACAAGCUAAAGUUACUGGCCAUAACAGCCAGAGGAAAAUUAAC
UUAAUUAUAUACAAAAACCUAUUCCUGUUGGCAUAGGCAAAUUGUAGAAGACAAAUCCAUGUAAGGAA
UAGGAAACCUAUUACUAGGUUCCAUUGUUCAAGGAGCUUUUUAAGCUCUUCAACGGUAAUAGUACCGU
UGGAAUCUGCCAUGGCUAAAAUUAAAGUUCCAAACAGAAAAACUAAUAUAAUAUUUAGUUCGUUUAGA
CCAGAAGAUCAGGAACUCUAGAAGAAUUCAGAUUUUUAACACGAGAGUAAACGUAAAAAGAAGGUUUU
ACAAGACUCACGUUAACAAUAUUGCAGCAGUACGCACACAAUCGAAGCGCAGUAAGGAUGGCUAGUGUA
ACUAGCAAGA

> Orf 6a Subgenomic
UCACAAGUAGCGAGUGUUAUCAGUGCCAAGAAAAGAAUAAUUUUCAUGUUCGUUUAAUCAAUCUCCAU
UGGUUGCUCUUCAUCUAAUUGAGAAUAUUUAUUCUCAGUUAGUGACUUAGAUAAAUUUUUAAUUAUG
AGGUUUAUGAUGUAAUCAAGAUUCCAAAUGGAAACUUUAAAAGUCCUCAUAAUAAUUAGUAAUAUCUC
UGCUAUAGUAACCUGAAAGUCAACGAGAUGAAACAUCUGUUGUCACUUACUGUACAAGCAAAGCAAUA
UUGUCACUGCUACUGGAAUGGUCUGUGUUUAAUUUAUAGUUGCCAAUCCUGUAGCGACUGUAUGCAG
CAAAACCUGAGUCACCUGCUACACGCUGCGAAGCUCCCAAUUUGUAAUAAGAAAGCGUUCGUGAUGUAG
CAACAGUGAUUUC

> Orf 7a subgenomic RNA
AGUUUAGGUGAAACUGAUCUGGCACGUAACUGAUAGACGUGUUUUACGCCGUCAGGACAAGCAAAAGC
AAAUUGAGUGCUAAAGCAAGUCAGUGCAAAUUUGUUAUCAGCUAGAGGAUGAAAUGGUGAAUUGCCCU

CGUAUGUUCCAGAAGAGCAAGGUUCUUUUAAAAGUACUGUUGUACCUCUAACACACUCUUGGUAGUGA
UAAAGCUCACAAGUAGCGAGUGUUAUCAGUGCCAAGAAAAGAAUAAUUUUCAUGUUCGUUUAAUCAAU
CUCCAUUGGUUGCUCUUCAUCUAAUUGAGAAUAUUUAUUCUCAGUUAGUGACUUAGAUAAAUUUUUA
AUUAUGAGGUUUAUGAUGUAAUCAAGAUUCCAAAUGGAACUUUAAAAGUCCUCAUAAUAAUUAGUAA
UAUCUCUGCUAUA

> Orf 7b Subgenomic RNA
GGGUCAUCAACUACAUAUGGUUGAUGUUGAGUACAUGACUGUAAACUACAUUCUUGGUGAAAUGCAGC
UACAGUUGUGAUGAUUCCUAAGAAAACAAGAAAUUUCAUGUUCGUUUAGGCGUGACAAGUUUCAUUA
UGAUCUUGCAGUUCAAGUGAGAACCAAAAGAUAAUAAGCAUAAUUAAAACAAGGAAUAGCAGAAAGGC
UAAAAAGCACAAAUAGAAGUCAAUUAAUGAAAGUUCAAUCAUUCUGUCUUUCUUUUGAGUGUGAAGCA
AAGUGUUAUAAACACUAUUGCCGCAACAAUAAGAAAAAUUGGAGAGUAAAGUUCUUGAACUUCCUCUU
GUCUGAUGAACAGUUUAGGUGAAACUGAUCUGGCACGUAACUGAUAGACGUGUUUUACGCCGUCAGGA
CAAGCAAAAGCAA

> Orf 8b SUbgenomic RNA
AGGAAACUGUAUAAUUACCGAUAUCGAUGUACUGAAUGGGUGAUUUAGAACCAGCCUCAUCCACGCAC
AAUUCAAUUAAAGGUGCUGAUUUUCUAGCUCCUACUCUAAUAUACCAUUUAGAAUAGAAGUGAAUAGG
ACACGGGUCAUCAACUACAUAUGGUUGAUGUUGAGUACAUGACUGUAAACUACAUUCUUGGUGAAAUG
CAGCUACAGUUGUGAUGAUUCCUAAGAAAACAAGAAAUUUCAUGUUCGUUUAGGCGUGACAAGUUUCA
UUAUGAUCUUGCAGUUCAAGUGAGAACCAAAAGAUAAUAAGCAUAAUUAAAACAAGGAAUAGCAGAAA
GGCUAAAAAGCACAAAUAGAAGUCAAUUAAUGAAAGUUCAAUCAUUCUGUCUUUCUUUUGAGUGUGAA
GCAAAGUGUUAU

> N gene subgenomic RNA
GAACGCCUUGUCCUCGAGGGAAUUUAAGGUCUUCCUUGCCAUGUUGAGUGAGAGCGGUGAACCAAGAC
GCAGUAUUAUUGGGUAAACCUUGGGGCCGACGUUGUUUUGAUCGCGCCCCACUGCGUUCUCCAUUCUG
GUUACUGCCAGUUGAAUCUGAGGGUCCACCAAACGUAAUGCGGGGUGCAUUUCGCUGAUUUUGGGGU
CCAUUAUCAGACAUUUUAGUUUGUUCGUUUAGAUGAAAUCUAAAACAACACGAACGUCAUGAUACUCU
AAAAAGUCUUCAUAGAACGAACAACGCACUACAAGACUACCCAAUUUAGGUUCCUGGCAAUUAAUUGUA
AAAGGUAAACAGGAAACUGUAUAAUUACCGAUAUCGAUGUACUGAAUGGGUGAUUUAGAACCAGCCUC
AUCCACGCACAA

NEGATIVE SENSE SUBGENOMIC RNAs of SARS-CoV

>S subgenomic

UUUGAGUCAAAGGGCAAAAAAUAGUCUUGAGUGAGGUGUAAAACAUCAGAACGAAAAAUGUCAUCAUU
AUAAUACACACCUCGCCGAGAGGAUGAGACUUUUUCCAUCUUUGGCUGUGGCUUUCUACUAAUAAUGC
CACAUCCUUCCUGUGCUUUAGCUAGACUGAAGAGCAGAGCAAAAAUUAAAACUUUCAUGUUCGUUUAG
UUGUUAACAAGAAUAUCACUUGAAACCACAACUCUAUUGUUUUCCCUAAUGAUAAGUCUACCAUUUUC
AAGAAGGGAAUAAAUCAUAUCAUUGAUUUGAUUCUCUUUUAAAGACAUAACAGCAGUCCCUCUUAACU
UGAGAGGAAAUUUGCUCAUGUCGAAAAGUGAAUAGGAAGACAAUUGAAUAGGAUUUGUGUUCCUCCA

>Orf3a Subgenomic

AAAAACAGCAAGAAGUGCAACGCCAACAACAAGCCAUCCGAAAGGGAGUGAGGCUUGUAGCGGUAUCGU
UGCUGUAGCAUGAACAGUACUUGCAGGAGAAGCAUUUUCAACUUUCGCUGGUUGGCGUGUGAUUGCU
CCCAAUGUGAAAAUGCUCAUAAACAAAUCCAUUAAGUUCGUUUAUGUGUAGUGUAGUUUGACUCCCUU
GAGCACUGGCUCAGAGUCGUCCUCAUCAAAUUUGCAGCAAGAACCGCAAGAGCAUGCACCCUUGAGGCA
ACUGCAACAGCUGGUCAUGCAACAAAGCAAGAUUGUAACCAUGACAAUGGCAAUUAGUCCGGCGAUGAA
GCCGAGCCAAACAUACCAAGGCCAUUUGAUGUAUUGCUCAUAUUUUCCAAGUUCUUGGAGGUCAAUGA

>E subgenomic

GUUUUACUAAACUCACGUUAACAAUAUUGCAGCAGUACGCACACAAUCGAAGCGCAGUAAGGAUGGCUA
GUGUGACUAGCAAGAAUACCACGAAAGCAAAAAAGAGAAGUACGCUAUUAACUAUUAACGUACCUGUU
UCUUCUGAAACGAAUGAGUACAUAAGUUCGUACUCACUUUCUUGUGCUUACAAAGGCACGCUAGUAGU
CGUCGUCGGCUCAUCAUAAAUUGGAUCCAUUGCUGGAUUUACAACUCCUGAAGAGCCGUCGAUUGUGU
GUAUUUGUACAUGGUCCACAUCUUUAACAAGCUUGCUAUAGAUGAAGAAUGUAGCAUUUUCAGCACCA
GUGUCGGUCGAUAGUUGUGUCGAUUCUAACUGGUAGUAGAUUUCAGUGAAAUAACCAUGUAUUACUA

>M Subgenomic

AGGAAAACAAGCUUUAUUAUGUACAGAAACCUGUUCCGGUUGGAAUAGGCAAACUGUAGUAGCAUAAU
CCAGGCAAGGAAAAUGAAACCUAUUACUAGAUUCCAUUGUUCCAGGAGUUGUUUAAGCUCCUCAACAG
UAAUUGUACCGUUGUCUGUCAUGAUAAGCAAUGUUAAAGUUCCAAACAGAAUAAUAAUAAUAGUUAGU
UCGUUUAGACCAGAAGAUCAGGAACUCCCUCAGAAGAGUUCAGAUUUUUAACACGUGAGUAAACGUAA
ACUGUUGGUUUUACUAAACUCACGUUAACAAUAUUGCAGCAGUACGCACACAAUCGAAGCGCAGUAAG
GAUGGCUAGUGUGACUAGCAAGAAUACCACGAAAGCAAAAAAGAGAAGUACGCUAUUAACUAUUAACG

>Orf6 subgenomic

AAUCUAACUCCAUAGGUUCUUCAUCAUCUAACUCAGAGUAUUUCUUCUUAGUUAGAGGCUUAAAUAAU
UGUCUCACUAUUGAACUUAUUAGUAUGUCAAGGUUCCAAAUGGCAACCCUGAAAGUCUUCAUAAUGAU
AAUCAAUAUCUCUGCUAUUGUAACCUGGAAGUCAACAAGAUGAAACAUCUGUUGUCACUUACUGUACU
AGCAAAGCAAUAUUGUCGUUGCUACCUGAGUGGUCUGUAUUUAAUUUAUAGUUCCCAAUUCGGUAGC
GGUUGUAUGCAGCAAAACCUGAAUCGUUGCCUACACGCUGCGAAGCUCCUAAUUUGUAAUAAGAAAGC
GUUCGUGAUGUAGCCACCGUAAUCUCUUUUGGCAGGUCCUUUAUGUCACAGCGCCCUAGGGAGUGUCC

>Orf 7 Subgenomic

AAAGCAAAAUGUGUGCUAAUGCAAGUUAGUGCAAAUUUGUUGUCAGCAAGAGGAUGAAAUGGUGAAU
UGCCCUCGUAGGUCCCUGACGGGCAAGGUUCUUUUAGUAGUACAGUGGUACCUCUAACACACUCCUGA
UAGUGAUAUAGCUCGCUGGAAGCAAGUGCAAUCAAUGUCAAGAAGAGAAUAAUUUUCAUGUUCGUUU
UAUGGAUAAUCUAACUCCAUAGGUUCUUCAUCAUCUAACUCAGAGUAUUUCUUCUUAGUUAGAGGCUU
AAAUAAUUGUCUCACUAUUGAACUUAUUAGUAUGUCAAGGUUCCAAAUGGCAACCCUGAAAGUCUUCA
UAAUGAUAAUCAAUAUCUCUGCUAUUGUAACCUGGAAGUCAACAAGAUGAAACAUCUGUUGUCACUUA

>Orf 8 subgenomic

AUUGUACAAGGCGAGCUGACUUUCUAGAUCCAAUUUUUAUAAACCAGUCCGAAUAGUAAUGUAUUGGA
CAUGGGUCUUCAAUUUGGUAGGGUUGAUUUUCACAACACUCUUGUAUGCUGCAUUCUUUAUGAAUGC
AGUAUACUGAUGUUAAGAGUCCAAAAACAAUGAGAAGUUUCAUGUUCGUUUAGACUUUGUUACAGGG
UUCUUCUAUAUCCUGGAGUUCAAGUGAAAACCAAAAUAUAAUAAGCAUUAUUAGAACAAGGAAUAGCA
GAAAGGCUAAAAAGCACAAAUAGAAGUCAAUUAAAGUGAGCUCAUUCAUUCUGUCUUUCUCUUAAUGG
UGAAGCAAAGUAUUAUAAAUACUAGAGCAGCAACAAUGAGAAAAAGUGGUGAAUAGAGCUCUUGGUGA

>N subgenomic

AGUAUUAUUGGGUAAACCUUGGGGACGGCGUUGCUUUGGCCGUGCACCACUGCGUCCUCCAUCCUGAU
UAUUGUCAGUUGAAUCUGAGGGUCCACCAAAUGUAAUGCGGGGGGCACUACGCUGGUUUUGGGGUCC
AUUAUCAGACAUUUUAGUUUGUUCGUUUAGAUGAAAUCUAGAACAACACGCACGUCAUGAUGCUCAAC
AAAGUCAUAAUCGUACGAGCAGCGCACAAUUAGACUACCUACUGGUGGUGCUUGACAGUUUAUCUCAA

GGGGUUCACAAGAGAUAGUGUAAUUGCCAAACAUUUCAUAAUGAAUCGGAAUUCUUUUGCCAUAAUCU
CCCUCGCACAAUUGUACAAGGCGAGCUGACUUUCUAGAUCCAAUUUUUAUAAACCAGUCCGAAUAGUAA

NEGATIVE SENSE SUBGENOMIC RNAs of MERS-CoV

```
>S gene sub-genomic RNA
GGUCUCCCUGAUAGGGAAAAAGACCUUGAUAAGUGAUAGUUAUGUUAGAAUAUGUACGGCCUUGAGGGUAUAUAA
UACCGUCAGCCUUAGAAACAUCAAUUGGCCUAGGCCAAGUUUUAUCAAAGAAAGUCUGUUGUAUAUCAACCUCAA
UACAAGCAGACUUAACAGAAUCUGGCCCUACAUCAACGUAACUUUCUGUAGGUGUUAACAAGAACAUCAGUAGAA
ACACUGAGUGUAUCAUUGUCACGGAUAAGUAACUUACCCUGCGACAGGAGAGAUAUUACGAGUUCGUUAAUUUGA
CUCUCCUUUAAUUGAAGAACUGGUGUUCCUUUUAAUUUUAAUUGAAACUUGGAUAAAUCAAAAAGUGAGUAAGUA
CUCAGAUUCAUAGGAGUGGAAUUUCUCCAAAAUAUAUAGUUGGCGUGCAUAGCACCACCAUCUAUAUUUUCUUUA

>Orf3 Subgenomic
GACAGAAACUGAUUGAGUACCAGUUGAUUCUGCAGAUGGGACGUCAAUUCGAAAAUUUGUAUAAAGACCAGCUGU
AUCAGCUUGGGCAGUUUUAAUACAAGCCCUAAGCAUGCAACCAGAAUAAUUCUGACAAUGCUCAGGUACAUAGAG
AGGUUUUGAAAAUGCAGUGACCAAAAGAGAGAGUGAGAACACUAACAAGAGAGUGGGUGGUCUUUGAACUCUCAU
UGAUAGUUCGUUAAUUAGUGAACAUGAACCUUAUGCGGCUCGAGGUCGUAUUCCUCGUAUCUAUCACAACAACGA
UUACACUUAAGUUUUCCCAUACAGUUUGUGCCACAACCAGUGCAGCACAGUAUGAAGAAGACGCAUAGAGCUAAG
GCAACAAGCCCAGCAAUGAAACCAAGCCAAAUGUACCACGGCCAUUUGUUGUAAUAAGUAUAAUUGCCAAGCUCU
> Orf 4a Subgenomic
AGGGAUUCCUCCAUCCUUAUGUAGCAACCAAGCGAUUCGCUGAGCUGCGUCCUGUUUGGCCAAAGCUUUUGUAGA
AUUAACAGCAGAUUCAGUAUAACCAGCAAAGGAUAGCUGACAGUUCCACAGCACAGGGUGCAAUGAAGUGCCAAC
UAAAGGUGUAUACUUAGCUGUGGGUUUAGGGAUAUACAAACAAGUAGUAUACGGUGAAUUAAGGUACUUCUGCCA
AAUUUGAUUAAGCAGAGACACGUAAUCCAUAGAGUUCGUUAAUUAACUGAGUAACCAACGUCAAAAAGAUUCACA
CUAGUAACAUGUUCGGUAGGACCAUCAUGAGUUGAAGUUGACUCACGAUCGACAGAAACUGAUUGAGUACCAGUU
GAUUCUGCAGAUGGGACGUCAAUUCGAAAAUUUGUAUAAAGACCAGCUGUAUCAGCUUGGGCAGUUUUAAUACAA
> Orf 5 Subgenomic
UGGCUAAUAAAAAGUUAUACAGAACAAGGAAAUAGAGAACCAUAGUUCUGAUAAUGCUGCGACGACAAGUAUCUU
GACGUAGCAGUAAUAAUAGGGGAAUGAGACACACAUUGACAGCUAAAGCAGCUACAUAGCCGCUAGCAGGAAUGU
AUGUGAAAACAAUAGAGUCAGUAGACUCAAUGCGAUGAAAUGCAGGAGAAACUGGGACUAGCUGGACGGGUUUAA
AUAAAGACGCCGAGAAAGCCAUAGUUCGUUAAAAUCCUGGAUGAUGUAAAAUGGGGUAAGAAUGAACAAUUUGUU
UAUUACCCUGAUUGGAAGACCGUAUGGAGACAACAUCUACGGCCAAAGGAGUAACAAACAUUUGUUGAGGAUAAG
UUCUAAAGCAUGGCAUUGUAAUGAUGUAAUUGCGAGUAACAUCAUCCAGUGCAUGCAAUUUAGAGAUUGACAUAU
> E gene Subgenomic RNA
ACAUUAUGAAGGAGUUCGUUAAACCCACUCGUCAGGUGGUAGAGGGGGUUUACUAUCCUGGAAUUUUACAUAGAC
UGAACGUCCAGUAUUAUACAAGUAUAAUGCGGGCUGAACUAACAGGGUAUUGAAGCCUGUCAUACAUUGCACACA
UAAUCUAGUAGCCGUAAGGAAAGCCAUACACACCAAGAGUGUUAUAGCACAUACUACGGUAAAAAUGAAAAAGUU
UACUAUGAACAACCCUAUUCGUUCUUGGACAAAGGGUAACAUAGUUCGUUUUCCAUAUGUCCAAAGAGAGACUAA
UGGAUUAGCCUCUACACGGGACCCAUAGUAGCGCAGAGCUGCUUAAACGAUAAGCGAGCUCGGGGCGAUUAUGUG
AAGAGGAACUGAAUCGCGCGUUGCAGGCACGAAAACAGUGGAAACAUUGCCGUUUAAGGAGAAGUCAGUUACAGA
> M gene SUbgenomic RNA

UUGGAUAAACGGCGCUAAAUAUUGAUAGCGCCAUGGAAGAUGGCCAUAGGAGCCAUAAAACAAACAUUUUAAAGA
CAUAGACAGUCAUACUACGGGAUGGGUAUCCAUACUGUAGUACGAUAGUAAUUAAGAGAAAGAUCAGGGACCAUG
CAAAGUUCCAGUCUUUAAUAAUGGCAAUAAUCUGCGCCUCAGUGAGUUGCGUCAUAUUAGACAUUAUGAAGGA
GUUCGUUAAACCCACUCGUCAGGUGGUAGAGGGGGUUUACUAUCCUGGAAUUUUACAUAGACUGAACGUCCAGUA
UUAUACAAGUAUAAUGCGGGCUGAACUAACAGGGUAUUGAAGCCUGUCAUACAUUGCACACAUAAUCUAGUAGCC
GUAAGGAAAGCCAUACACACCAAGAGUGUUAUAGCACAUACUACGGUAAAAAUGAAAAAGUUUACUAUGAACAAC
> N gene subgenomic RNA

UUCCAUUCCCGGUAUUAAUUUUUCUGUCCUGUCUCCGCCAAUACCCAGCAUUUUGCGCAGGGGUGGAAUUGGCAU
UAAGAGGUACACCCUGCCCAGGUGGAAAGGUAAGAGGGACUUUCCCGUGUUGGGUAAGCCCAGUGUACCAAGAGA
CAGUGUUAUUUGGUGCAGCUCGUGGUUUUGGAUUACGUCCUCUACCUCGAGACAGGUUUGUAUUUGUUAUAUCAU
UGUUAUCGGCAAAGGAAACAGCACGAGGUGCAGCAGGGGAUGCCAUAACAAUGAAAUUGAGAUUCGUUAAAAUCA
AUUAAGAUACUCUUACUAAAGAGCCUAAGCUCGAAGCAAUGCAAGUUCAAUAUCCGCCGUAAUAGGCGGACUCCU
GUAAUUACCUGCCUUAUAUCUAUGGUAAAUGGCAACGCCGGAAUUAGUUCCGUAGCUUUGCCGCUUCACCAUUUU
```

<u>Sequences used for secondary structure prediction of SARS-CoV-2 subgenomic RNAs by using Vienna RNA webserver:</u>

Specific length of aligned RNA sequence was used for secondary structure prediction

>S Subgenomic

AACUGAGGAUCUGAAAACUUUGUCAGGGUAAUAAACACCACGUGUGAAAGAAUUAGUGUAUGCAGGGG
GUAAUUGAGUUCUGGUUGUAAGAUUAACACACUGACUAGAGACUAGUGGCAAUAAAACAAGAAAAACA
AACAUUGUUCGUUUAGUUGUUAACAAGAACAUCACUAGAAAUAACAACUCUGUUGUUUUCUCUAAUUA
UAAGUCUACCUUUACUAAGAAGAGAUAAAAUCAUAUCAUUGAUUUGACCUUCUUUUAAAGACAUAACA
GCAGUACCCCU

>Orf3a Subgenomic

AGCGCGAACAAAAUCUGAAGGAGUAGCAUCCUUGAUUUCACCUUGCUUCAAAGUUACAGUUCCAAUUG
UGAAGAUUCUCAUAAACAAAUCCAUAAGUUCGUUUAUGUGUAAUGUAAUUUGACUCCUUUGAGCACUG
GCUCAGAGUCGUCUUCAUCAAAUUUGCAGCAGGAUCCACAAGAACAACAGCCCUUGAGACAACUACAGC
AACUGGUCAUACAGCAAAGCAUAAUUGUCACCAUUACUAUGGCAAUCAAGCCAGCUAUAAAACCUAGCC
AAAUGUACCAU

>E subgenomic

AGAAUACCACGAAAGCAAGAAAAAGAAGUACGCUAUUAACUAUUAACGUACCUGUCUCUUCCGAAACGA
AUGAGUACAUAAGUUCGUACUCAUCAGCUUGUGCUUACAAAGGCACGCUAGUAGUCGUCGUCGGUUCA
UCAUAAAUUGGUUCCAUUACUGGAUUAACAACUCCGGAUGAACCGUCGAUUGUGUGAAUUUGGACAUG
UUCUUCAGGCUCAUCAACAAUUUUAUUGUAGAUGAAGAAGGUAACAUGUUCAACACCAGUGUCUGUAC
UCAAUUGAGU

>M subgenomic

UAGGAAACCUAUUACUAGGUUCCAUUGUUCAAGGAGCUUUUUAAGCUCUUCAACGGUAAUAGUACCGU
UGGAAUCUGCCAUGGCUAAAAUUAAAGUUCCAAACAGAAAAACUAAUAUAAUAUUUAGUUCGUUUAGA
CCAGAAGAUCAGGAACUCUAGAAGAAUUCAGAUUUUUAACACGAGAGUAAACGUAAAAAGAAGGUUUU
ACAAGACUCACGUUAACAAUAUUGCAGCAGUACGCACACAAUCGAAGCGCAGUAAGGAUGGCUAGUGUA
ACUAGCAAGA

> Orf6a Subgenomic

AGGUUUAUGAUGUAAUCAAGAUUCCAAAUGGAAACUUUAAAAGUCCUCAUAAUAAUUAGUAAUAUCUC
UGCUAUAGUAACCUGAAAGUCAACGAGAUGAAACAUCUGUUGUCACUUACUGUACAAGCAAAGCAAUA
UUGUCACUGCUACUGGAAUGGUCUGUGUUUAAUUUAUAGUUGCCAAUCCUGUAGCGACUGUAUGCAG
CAAAACCUGAGUCACCUGCUACACGCUGCGAAGCUCCCAAUUUGUAAUAAGAAAGCGUUCGUGAUGUAG
CAACAGUGAUUUC

>Orf7a subgenomic

CGUAUGUUCCAGAAGAGCAAGGUUCUUUUAAAAGUACUGUUGUACCUCUAACACACUCUUGGUAGUGA
UAAAGCUCACAAGUAGCGAGUGUUAUCAGUGCCAAGAAAAGAAUAAUUUUCAUGUUCGUUUAAUCAAU
CUCCAUUGGUUGCUCUUCAUCUAAUUGAGAAUAUUUAUUCUCAGUUAGUGACUUAGAUAAAUUUUUA
AUUAUGAGGUUUAUGAUGUAAUCAAGAUUCCAAAUGGAAACUUUAAAAGUCCUCAUAAUAAUUAGUAA
UAUCUCUGCUAUA

>Orf7b subgenomic

UGAUCUUGCAGUUCAAGUGAGAACCAAAAGAUAAUAAGCAUAAUUAAAACAAGGAAUAGCAGAAAGGC
UAAAAAGCACAAAUAGAAGUCAAUUAAUGAAAGUUCAAUCAUUCUGUCUUUCUUUUGAGUGUGAAGCA
AAGUGUUAUAAACACUAUUGCCGCAACAAUAAGAAAAAUUGGAGAGUAAAGUUCUUGAACUUCCUCUU
GUCUGAUGAACAGUUUAGGUGAAACUGAUCUGGCACGUAACUGAUAGACGUGUUUUACGCCGUCAGGA
CAAGCAAAAGCAA

> Orf 8b subgenomic

ACACGGGUCAUCAACUACAUAUGGUUGAUGUUGAGUACAUGACUGUAAACUACAUUCUUGGUGAAAUG
CAGCUACAGUUGUGAUGAUUCCUAAGAAAACAAGAAAUUUCAUGUUCGUUUAGGCGUGACAAGUUUCA
UUAUGAUCUUGCAGUUCAAGUGAGAACCAAAAGAUAAUAAGCAUAAUUAAAACAAGGAAUAGCAGAAA
GGCUAAAAAGCACAAAUAGAAGUCAAUUAAUGAAAGUUCAAUCAUUCUGUCUUUCUUUUGAGUGUGAA
GCAAAGUGUUAU

>N subgenomic

GUUACUGCCAGUUGAAUCUGAGGGUCCACCAAACGUAAUGCGGGGUGCAUUUCGCUGAUUUUGGGGU
CCAUUAUCAGACAUUUUAGUUUGUUCGUUUAGAUGAAAUCUAAAACAACACGAACGUCAUGAUACUCU
AAAAAGUCUUCAUAGAACGAACAACGCACUACAAGACUACCCAAUUUAGGUUCCUGGCAAUUAAUUGUA
AAAGGUAAACAGGAAACUGUAUAAUUACCGAUAUCGAUGUACUGAAUGGGUGAUUUAGAACCAGCCUC
AUCCACGCACAA

Supplementary Figures: Secondary structure of negative sense subgenomic RNAs (sequences are provided in page number 3-4)

Figure 1: E subgenomic

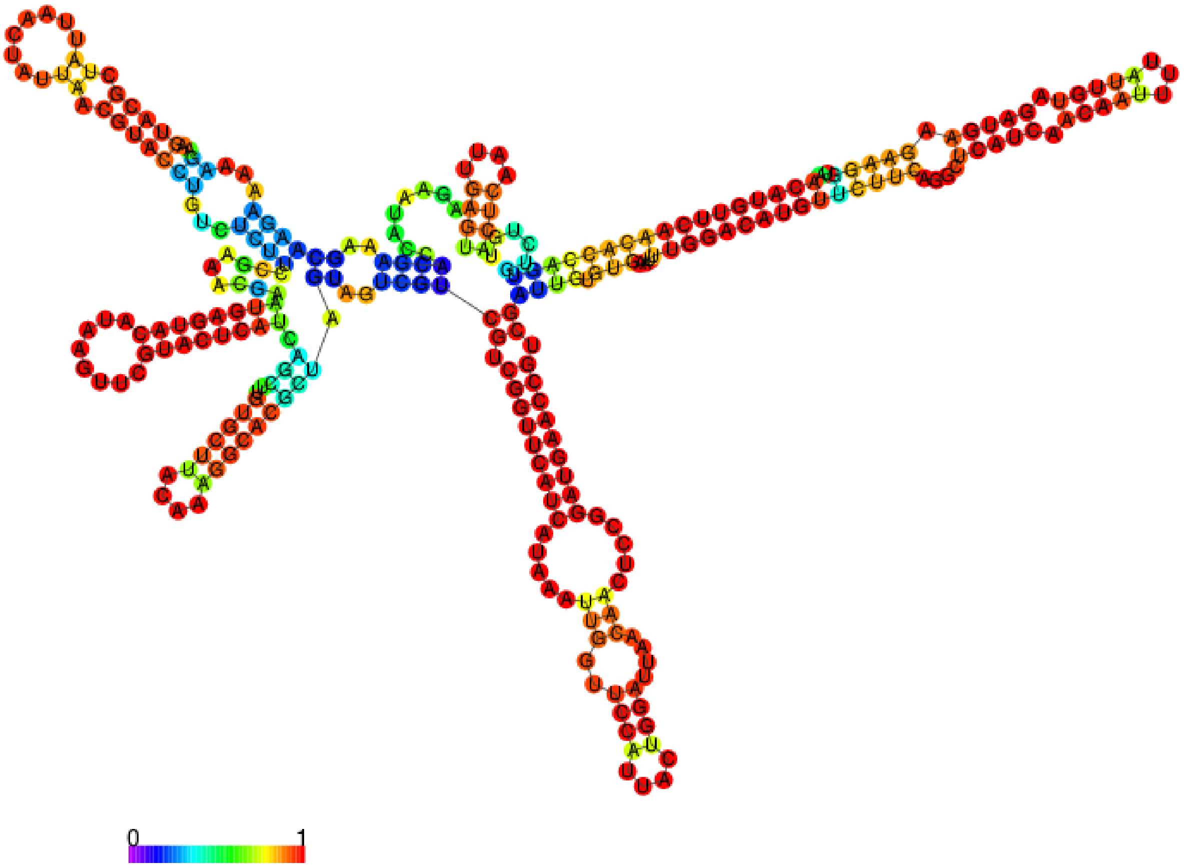

Figure 2: M subgenomic

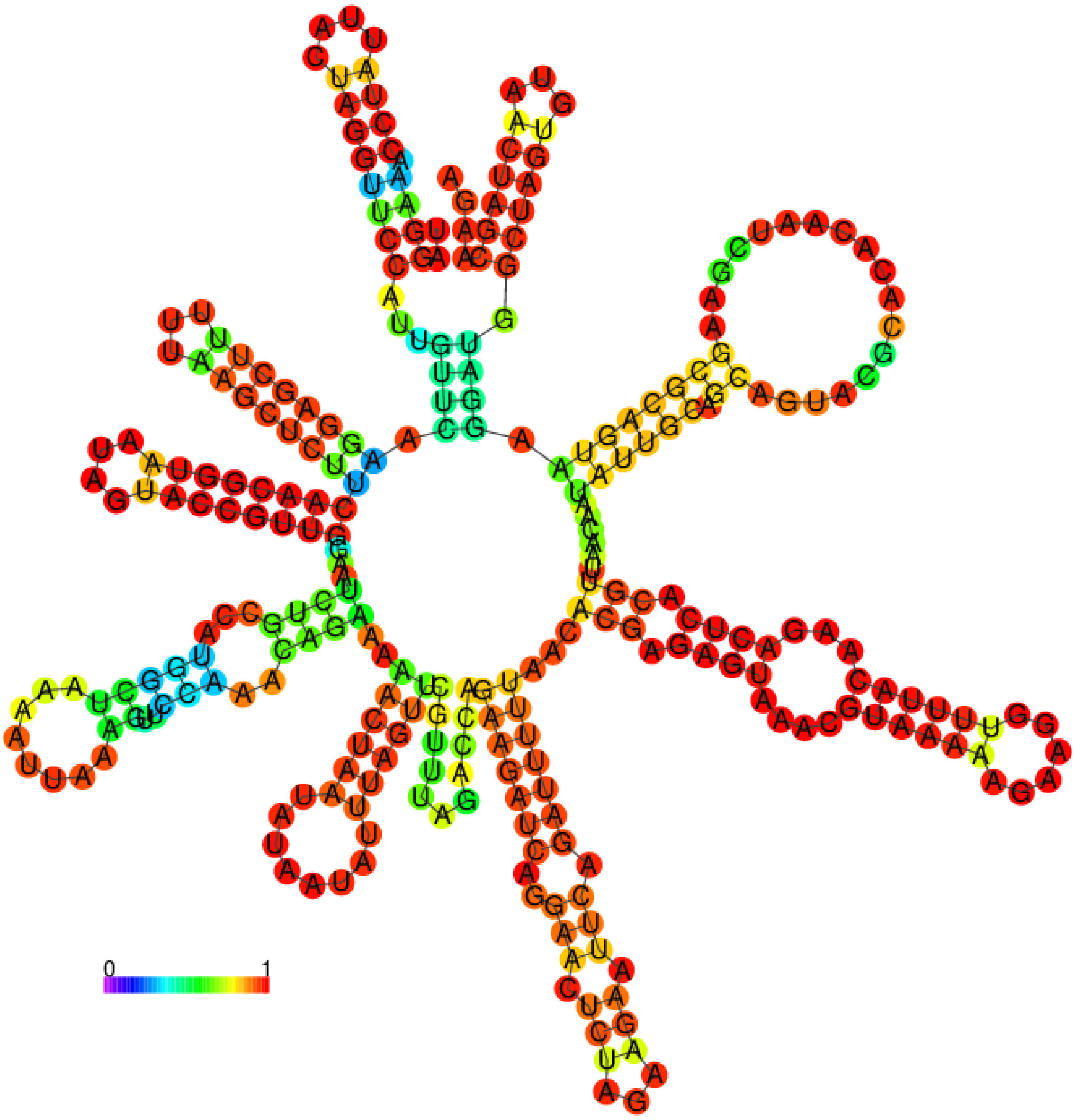

Figure 3: N subgenomic

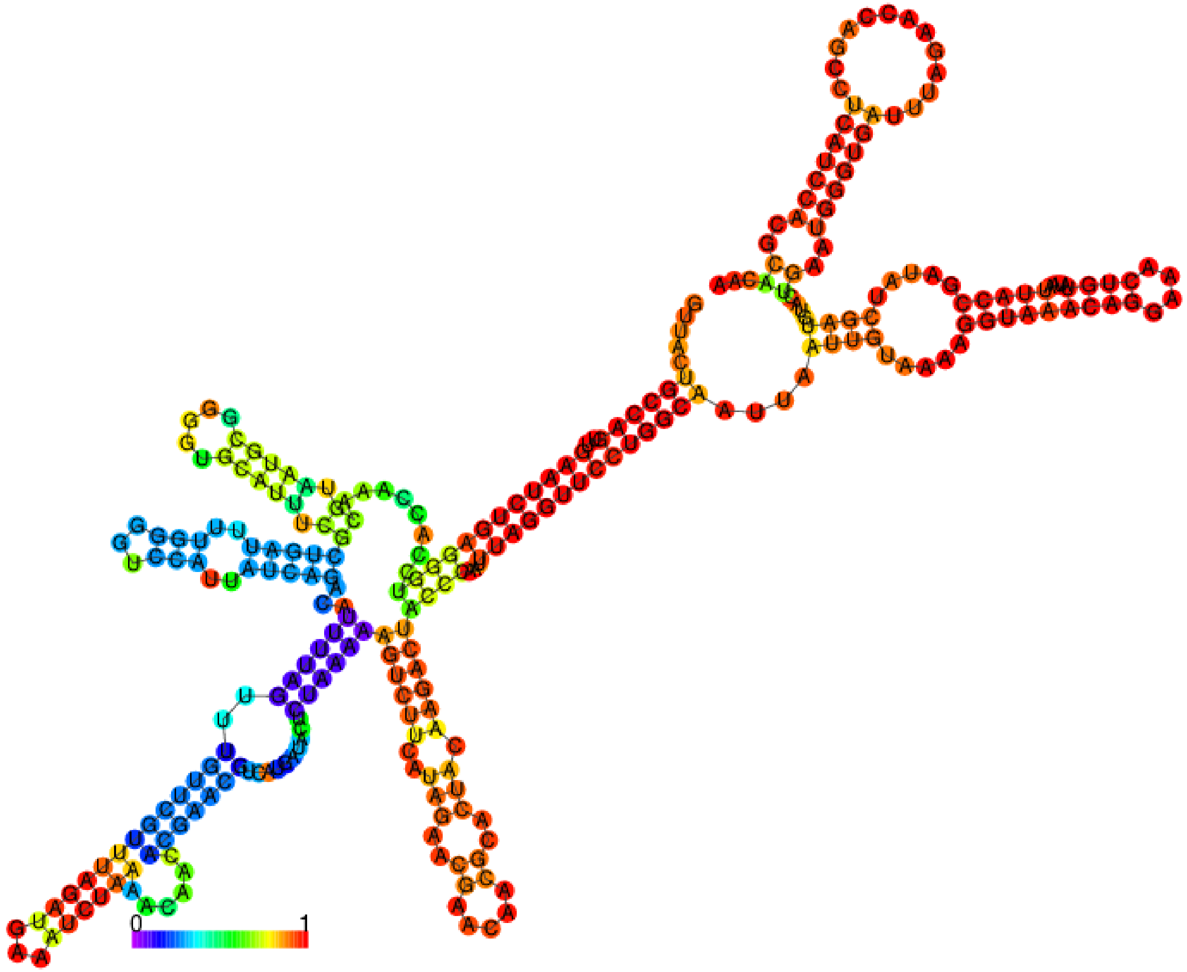

Figure 4: Orf3a Subgenomic

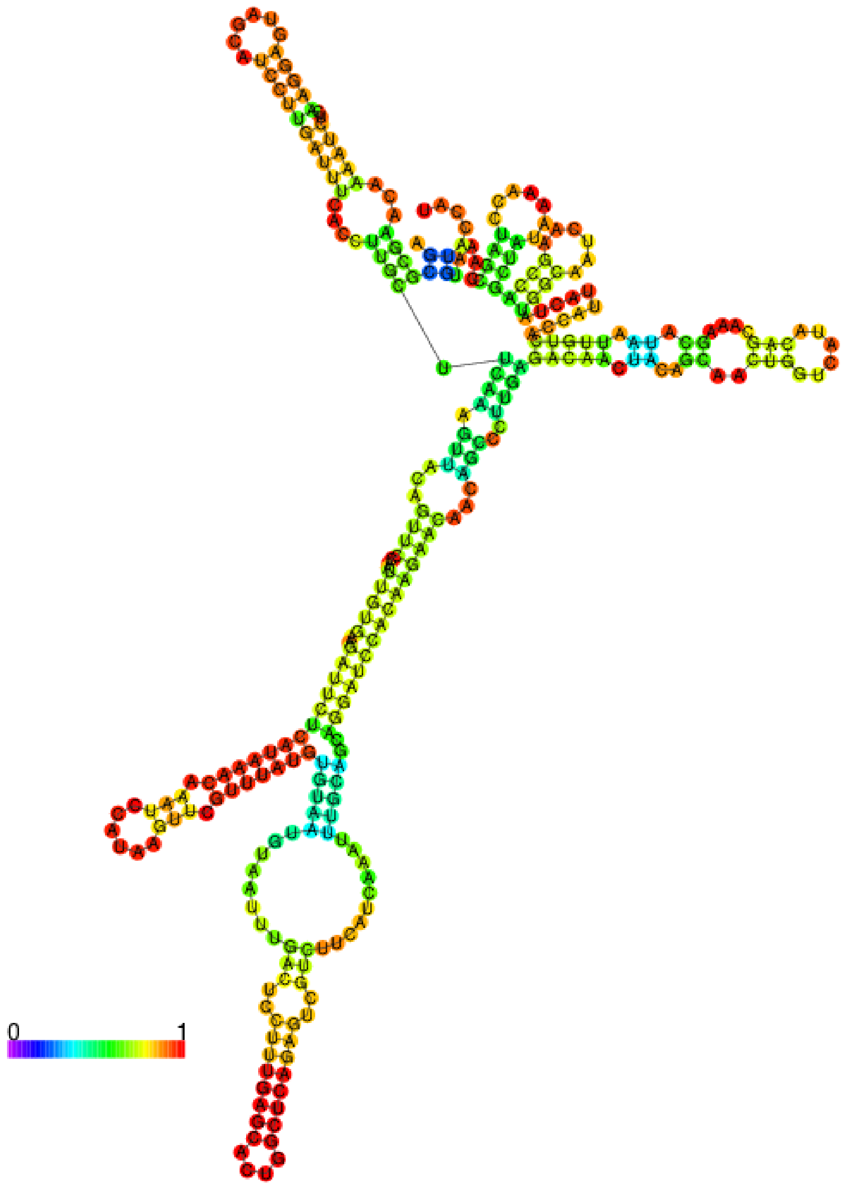

Figure 5: Orf 6 Subgenomic

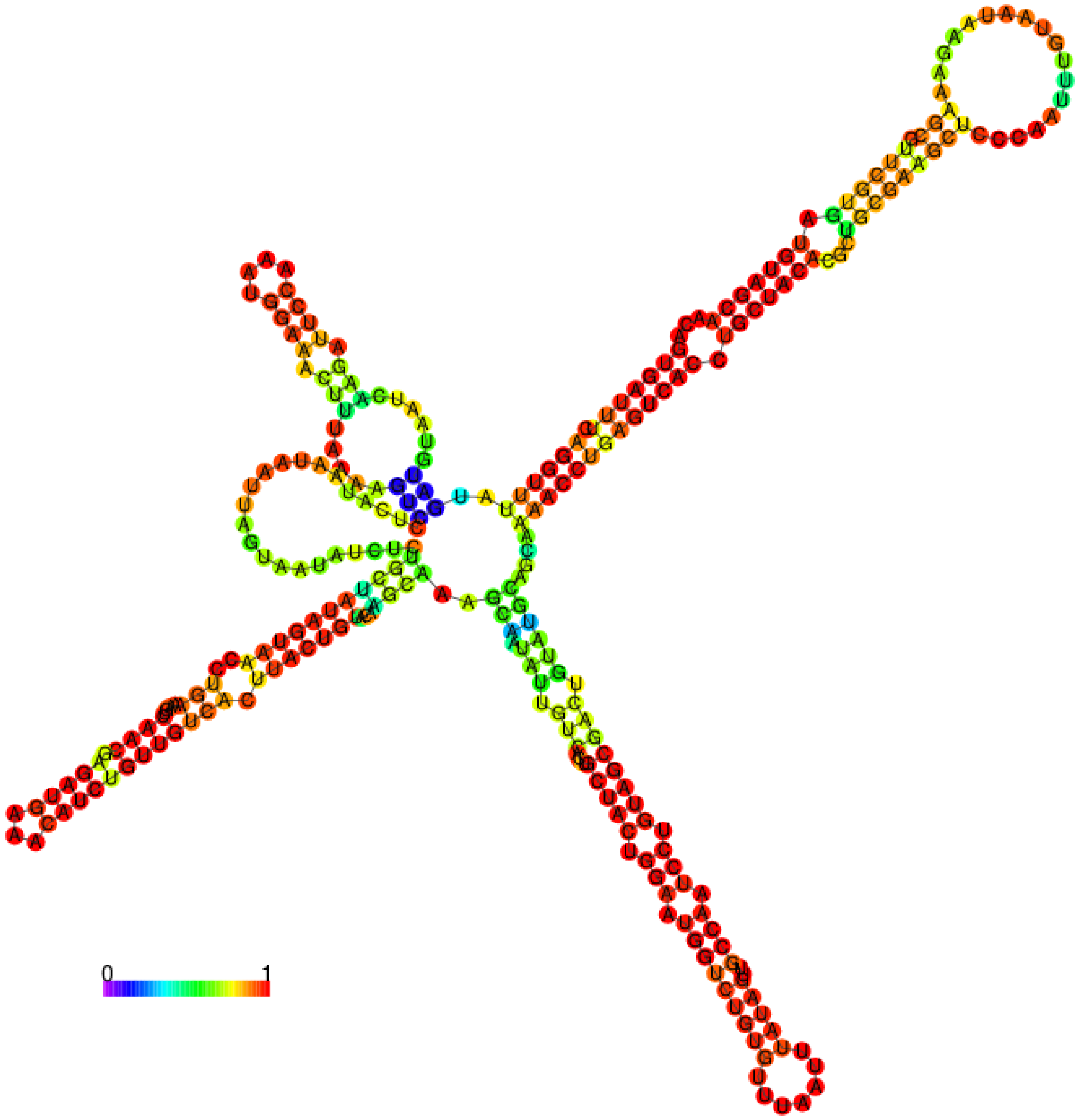

Figure 6: Orf 7a subgenomic

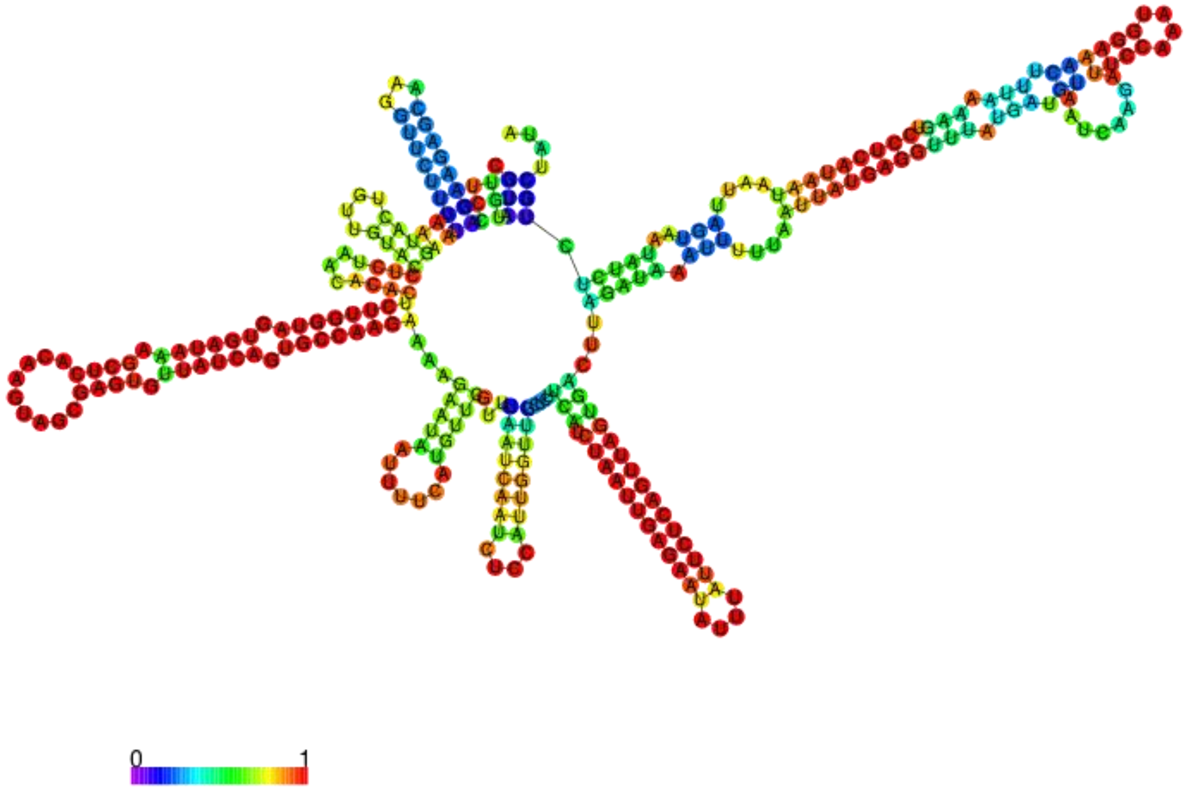

Figure 7: Orf 7b subgenomic

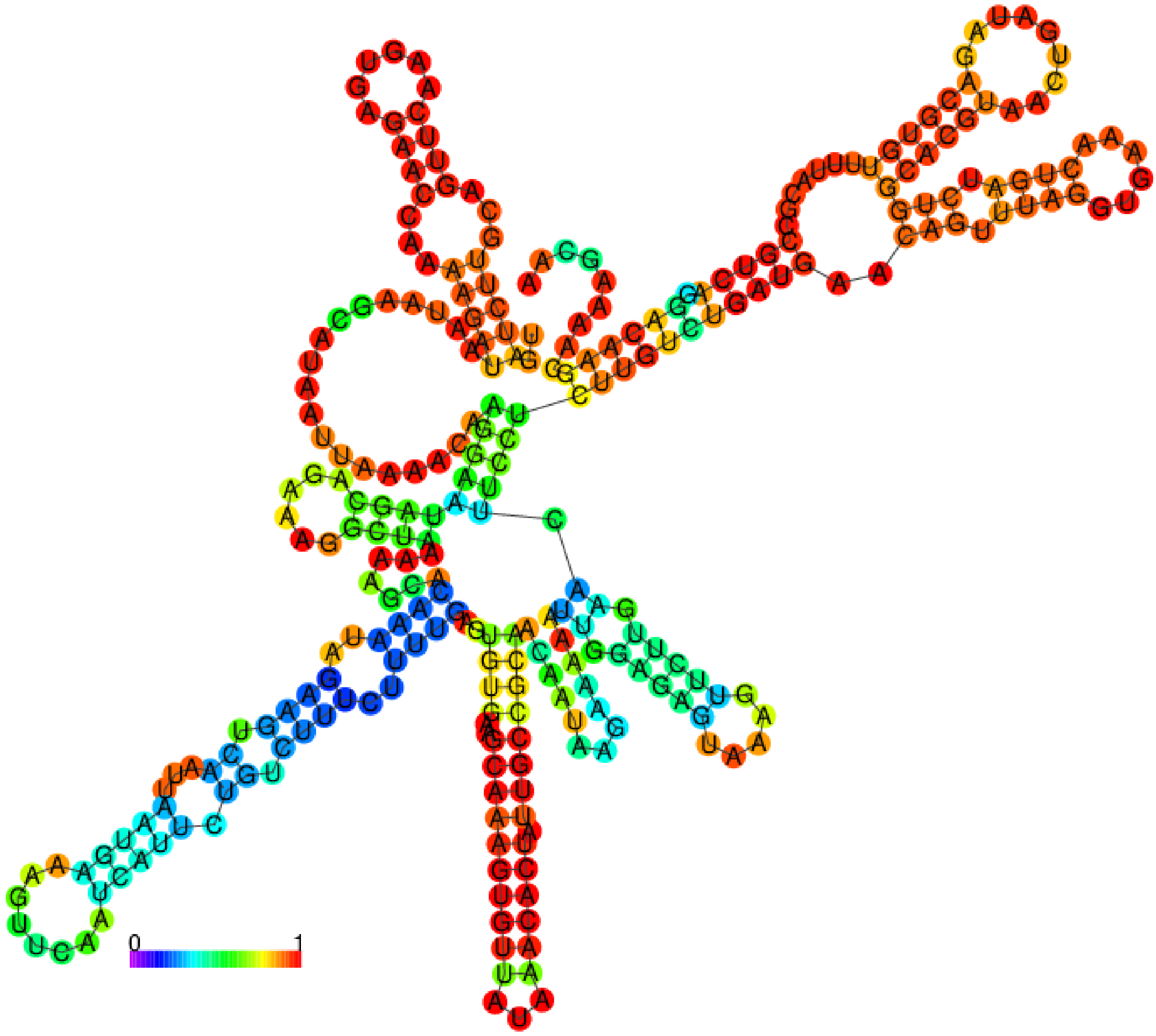

Figure 8: Orf 8 subgenomic

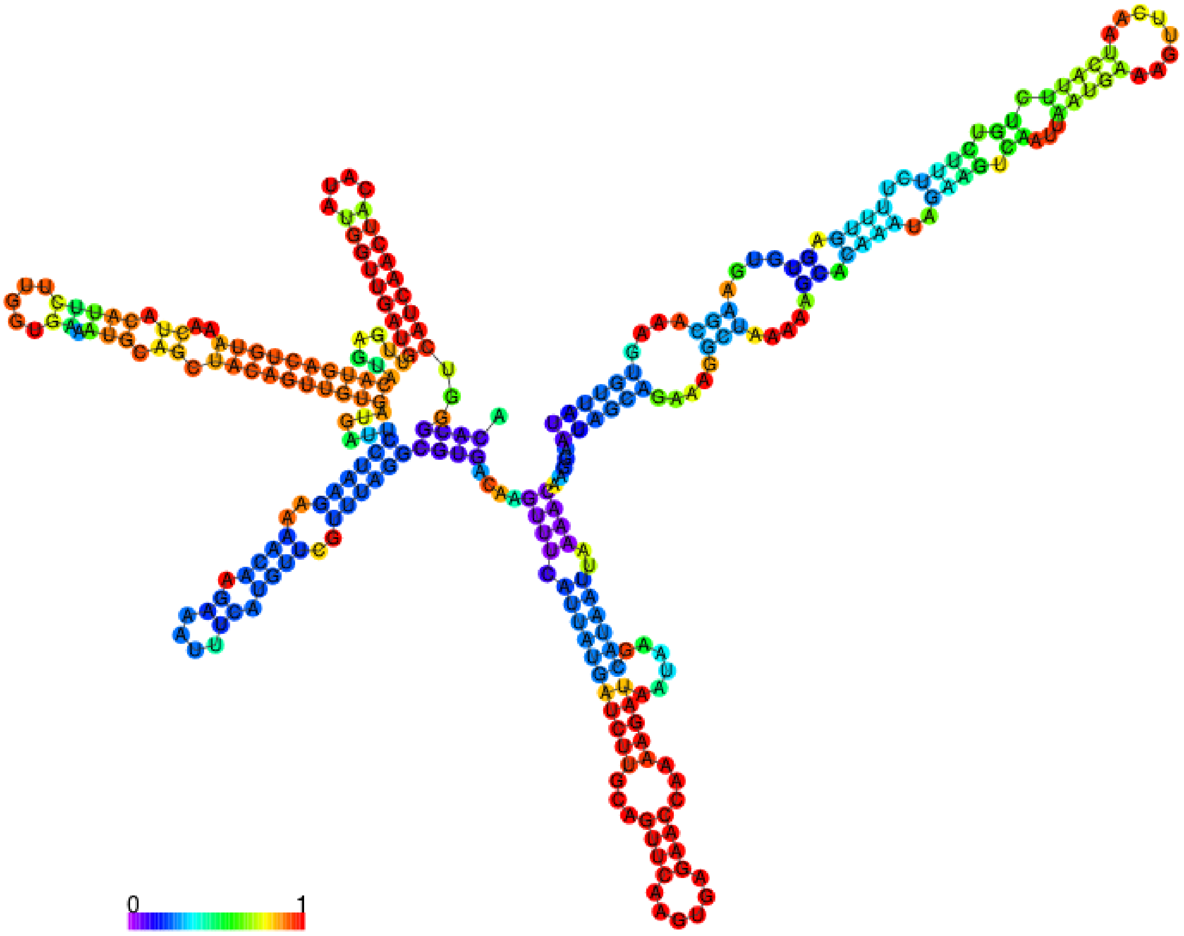

Figure 9: Orf S subgenomic

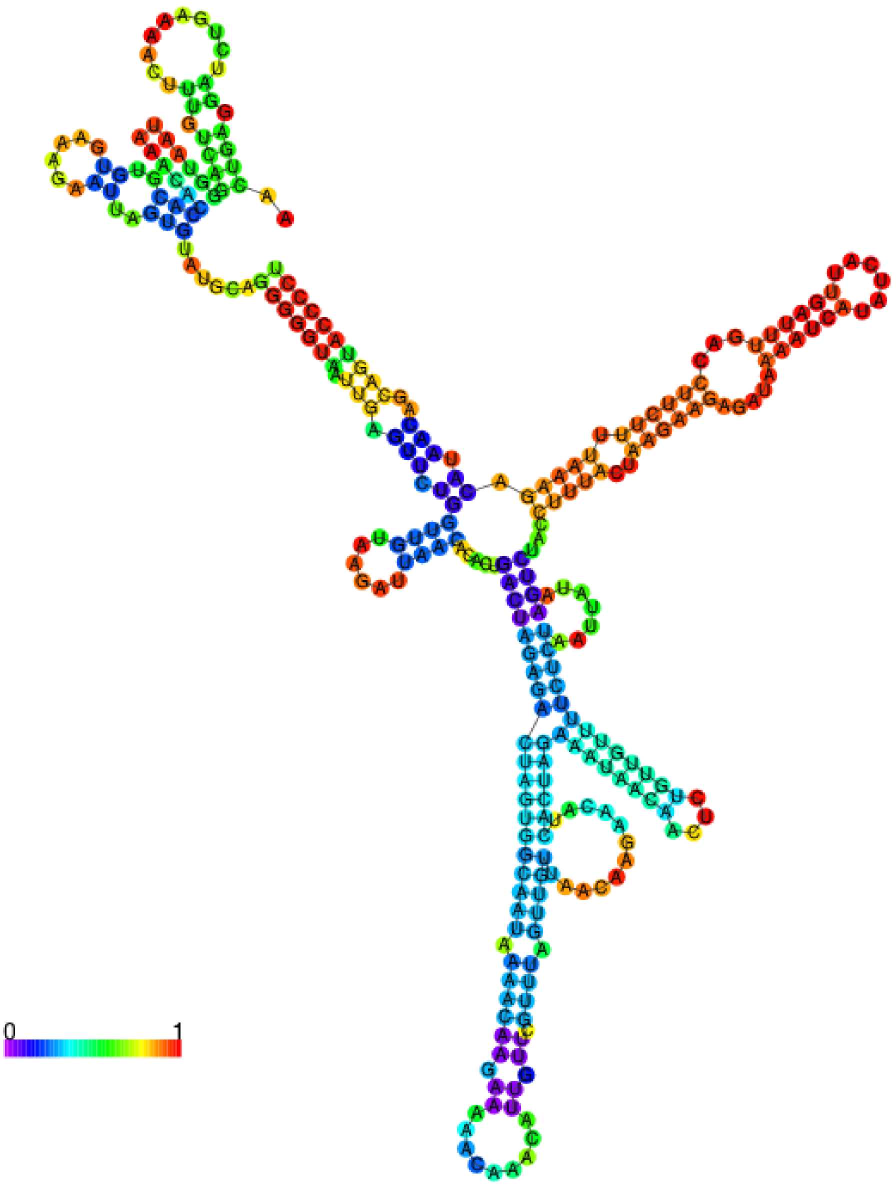
0
1
